# From Acid Alpha-Glucosidase Deficiency to Autophagy: Understanding the Bases of POMPE Disease

**DOI:** 10.3390/ijms241512481

**Published:** 2023-08-05

**Authors:** Valentina Sánchez-Porras, Johana Maria Guevara-Morales, Olga Yaneth Echeverri-Peña

**Affiliations:** Instituto de Errores Innatos del Metabolismo, Facultad de Ciencias, Pontificia Universidad Javeriana, Carrera 7 # 43-82, Ed. 54, Lab 303A, Bogotá 110231, Colombia; sanchez.valentina@javeriana.edu.co

**Keywords:** acid alpha-glucosidase, autophagy, glycogen storage disease type II, interaction network, Pompe disease

## Abstract

Pompe disease (PD) is caused by mutations in the *GAA* gene, which encodes the lysosomal enzyme acid alpha-glucosidase, causing lysosomal glycogen accumulation, mainly in muscular tissue. Autophagic buildup is considered the main factor affecting skeletal muscle, although other processes are also involved. Uncovering how these mechanisms are interconnected could be an approximation to address long-lasting concerns, like the differential skeletal and cardiac involvement in each clinical phenotype. In this sense, a network reconstruction based on a comprehensive literature review of evidence found in PD enriched with the STRING database and other scientific articles is presented. The role of autophagic lysosome reformation, PGC-1α, MCOLN1, calcineurin, and Keap1 as intermediates between the events involved in the pathologic cascade is discussed and contextualized within their relationship with mTORC1/AMPK. The intermediates and mechanisms found open the possibility of new hypotheses and questions that can be addressed in future experimental studies of PD.

## 1. Introduction

Pompe disease (PD), or glycogen storage disease type II, is an autosomal recessive inherited metabolic disease caused by mutations in acid alpha-glucosidase (GAA; EC 3.2.1.20), a lysosomal enzyme involved in lysosomal glycogen catabolism [1,2].

Classic infantile-onset PD (IOPD) is the most severe phenotype. These patients have low GAA residual activity (<1%) and develop symptoms like cardiomegaly, hypotonia, hepatomegaly, and respiratory failure within the first year of life [3]. Additionally, a non-classic IOPD has been described in which cardiomyopathy is less severe. On the other hand, patients with the late-onset type (LOPD) present higher residual enzyme activity (10–40%), and their clinical debut usually occurs beyond childhood. The main symptoms are progressive muscular weakness and respiratory failure but without cardiomyopathy [3,4,5,6].

In 2006, a therapy with a human recombinant enzyme was approved (Myozyme^®^/Lumizyme^®^), which proved to reverse cardiac abnormalities and extend IOPD patients’ lifespan, but it has a limited effect on skeletal muscle. Additionally, long-term survivors develop a new phenotype with symptoms not attributed to PD before [7,8,9,10].

It has been demonstrated that LOPD models and patients develop large tissue areas where autophagy is overinduced but not completed; thus, lysosomes and autophagosomes with undigested content are accumulated, which is known as autophagic buildup (AuP-BU) [11]. This buildup not only prevents the recombinant enzyme from reaching the lysosome [12] but also affects the structure of the sarcomeres, making them incapable of contracting [7]. Therefore, AuP-BU is considered the main factor compromising the functionality of skeletal muscle in PD [12,13,14].

The AuP-BU observed in PD has been attributed mainly to a disruption in the AMPK (AMP-activated protein kinase) and mTORC1 (mechanistic target of rapamycin complex 1) signaling pathways, two master regulators of autophagy [15,16]. Nevertheless, besides AuP-BU, unbalance between protein synthesis and degradation [13], energy metabolism disturbances [17], and anomalies in calcium homeostasis and in mitochondrial functioning have been reported [8,18,19]. Although understanding of PD has grown, it remains unclear how all these processes and molecular elements are connected. Traditionally, studies addressing such issue have been experimental; however, an alternative method to elucidate pathological mechanisms is the design of in silico interaction networks. These networks integrate what is already known and provide insights into interactions and molecular elements that have not been fully considered in experimental studies without presenting limitations due to resource availability like patients or laboratory equipment. Hence, they can generate new hypotheses and direct future investigations.

Considering the above, the purpose of this investigation was to identify knowledge gaps related to molecular mechanisms involved in PD through the construction of an interaction network that incorporates experimental evidence in PD, along with information from the STRING database and literature external to the context of the disease. The network summarizes current knowledge about PD pathophysiology, revealing that although most literature describes the role of mTORC1/AMPK and autophagy, there is evidence of alterations in eight cellular processes involving more than 50 molecular intermediaries. In fact, some processes, like energy metabolism and ER stress, have been poorly explored. Thus, network construction and enrichment allowed for the identification of potential roles of new molecular intermediates and processes that have not been previously considered in the context of PD, such as PGC-1α and the autophagic reformation of lysosomes, resulting in the proposal of novel molecular mechanisms that can be addressed in future studies.

## 2. Results and Discussion

### 2.1. Network Characteristics

**Figure 1:**
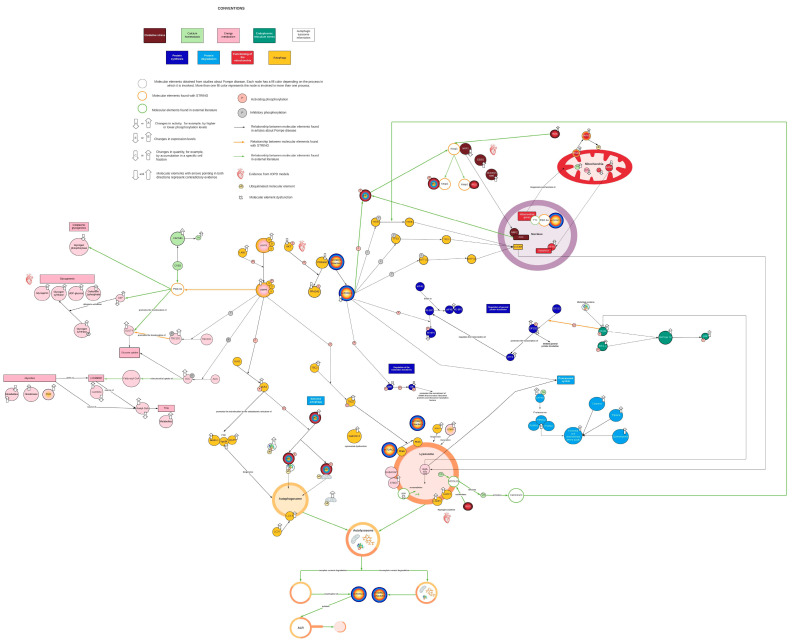
Complete view of the network. Each color represents a different cellular process. Yellow corresponds to autophagy, pink corresponds to energy metabolism, aquamarine corresponds to endoplasmic reticulum stress, light green corresponds to calcium homeostasis, red wine corresponds to oxidative stress, red corresponds to mitochondrial function, dark blue corresponds to protein synthesis, and light blue corresponds to protein breakdown. Black lines indicate interactions reported in the PD literature. Orange lines represent interactions identified by STRING. Green lines represent interactions supported by the literature external to the PD context. Open orange circles represent nodes found in STRING. Green open circles represent nodes identified by literature enrichment. Created with Lucidchart^®^ (https://www.lucidchart.com/pages/ accessed on 8 June 2023).

The initial network based on available PD information (see Section 3.1) included 68 molecular intermediaries (nodes) that were organized in eight cellular processes: selective and nonselective autophagy (24 nodes), energy metabolism (22 nodes), endoplasmic reticulum (ER) stress (4 nodes), calcium homeostasis (3 nodes), oxidative stress (5 nodes), functioning of the mitochondria (7 nodes), and synthesis and degradation of proteins (7 and 8 nodes, respectively) (Figure 1). Functional or physical interactions among nodes described by PD research articles are represented as solid black lines in Figure 1. Within this initial network, molecular mechanisms explaining the changes reported in some elements of energy metabolism (8 out of 22 nodes), oxidative stress, ER stress, and calcium homeostasis were not clearly identified (Figure 1). Thus, such processes were “isolated” in the network, revealing some knowledge gaps that require further research. As a theoretical approach to identify possible intermediaries that could help to bridge those gaps, interactions of nodes included in the network were explored in STRING. This enrichment provided two new potential nodes (PGC-1α and Keap1, represented as open orange circles in Figure 1) and three new potential interaction between nodes not linked in the initial network (solid orange lines Figure 1). To complete the enrichment process, eight more articles (three reviews and five research articles) were consulted (see Section 3.2.2), which provided one new cellular process (autophagic lysosome reformation (ALR)), four additional nodes (YY1, calcineurin, MCOLN1, and Spin, represented as open green circles Figure 1), and novel interactions between existing nodes (solid green lines Figure 1). Therefore, the final network comprises 74 nodes and nine cellular processes (Figure 1 and Table S3).

### 2.2. AMPK and mTORC1 as Origin Nodes

**Figure 2:**
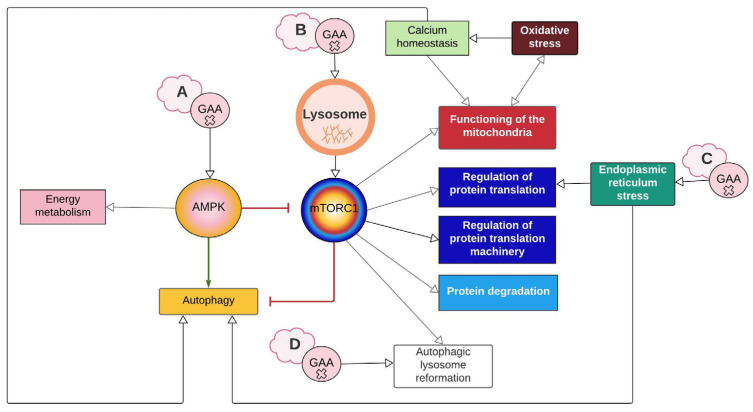
Summary of interaction between cellular processes represented on the network. The lettered clouds represent the different hypotheses that exist around how GAA mutations cause disruption in autophagy. (**A**) GAA deficiency reduces glucose availability, thus activating the AMPK pathway. (**B**) Accumulation of glycogen damages the lysosome, which prevents mTORC1 from activating. (**C**) Misfolded GAA triggers ER stress. (**D**) GAA defect inhibits mTORC1 reactivation and ALR initiation. White arrows imply a relationship between the processes. The green arrow represents activation, and the red arrows represent inhibition. Created with Lucidchart^®^ (https://www.lucidchart.com/pages/ accessed on 8 June 2023).

AMPK and mTORC1, master regulators of autophagy, are among the most researched molecular elements in PD. The network shows that disruptions on both pathways either directly or indirectly compromise all nine of the identified cellular processes (Figure 1 and Figure 2). According to the evidence found in PD, their contribution to the pathologic cascade is mainly related to AuP-BU, since they activate and inhibit autophagy depending on nutrient cues. Experimental evidence found in tissues of LOPD patients points out that AMPK activity is increased, while mTORC1 basal activity is decreased [15]. In this regard, there are two hypotheses: One is that GAA deficiency limits the glucose available within the cell; therefore, AMPK becomes activated and inhibits mTORC1 through TSC2 (tuberous sclerosis complex 2) (Figure 1 and Figure 2A). The second is that excessive glycogen accumulation damages the lysosome and prevents mTORC1 from being activated because lysosomes act as mTORC1 activation platforms (Figure 2B) [20].

Besides the abovementioned uncertainties regarding the events linking GAA deficiency and disruption of mTORC1/AMPK function, the available evidence points out that AuP-BU compromises skeletal muscle functionality, but this appears not to be the case for cardiac muscle. Moreover, there is recent evidence of AuP-BU in smooth muscle [21]. Raval et al. (2015) mentioned that there is hypertrophy rather than cardiac atrophy during PD. Experimental evidence seems to agree with this perspective, since it shows that there is no accumulation of autophagic markers like LC3 (microtubule-associated protein 1A/1B-light chain 3) and SQSTM1/p62 (sequestosome 1) in iPSC-derived cardiomyocytes from IOPD patients [22]. Furthermore, Sato et al. (2016) did not evidence a significant difference in *p*-AMPK (activated form) in iPSC-derived cardiomyocytes from LOPD patients, nor in cardiomyocytes from GAA knockout (KO) mice (Table S2). Therefore, current research suggests that autophagy does not play a key role in IOPD pathophysiology, at least in the early stages of the cardiomyopathy [22,23]. However, the pathologic cascade is not limited to autophagy. As discussed in the following sections, both AMPK and mTORC1 are key molecular elements where most processes that participate in PD converge.

### 2.3. Energy Metabolism Alterations Mediated by AMPK and PGC-1α

Pompe disease is an inherited metabolic disorder of glycogen metabolism, an important fuel source, therefore implicating energetic alterations that some authors have correlated with clinical manifestations observed mainly in LOPD patients [9]. Unfortunately, during network construction, we identified that research focused on this metabolism and its impact on energy availability in PD pathophysiology is still limited and unclear. In fact, only four included nodes correspond to glycogen metabolism, and for four nodes (involved in glycolysis and beta-oxidation), the evidence is contradictory (Figure 1).

Regarding the hypothesis of AMPK activation and mTORC1 inhibition due to the lack of glucose (Figure 2A), two aspects should be considered. One is the role of lysosomal glycogen degradation, since there is a cytoplasmic pathway for glycogen degradation, and this route could provide the cell with enough glucose to supply its needs. The second is that the evidence found in GAA-KO mice suggests that cells activate mechanisms to obtain glucose, such as the increase in GLUT4, along with the inhibitory phosphorylation of ACC and TBC1D1, which promote GLUT4 translocation to the cytoplasmic membrane (Figure 1) [24]. Therefore, AMPK activation and mTORC1 inhibition would not be permanent, as seems to be the case in PD, unless other pathways like ARL, which will are discussed later, are contemplated.

The evidence from metabolic profiles does not seem conclusive either. Meena et al. (2020) suggest that there is a shift from glycolysis to β-oxidation as the primary source of acetyl CoA based on a decrease in the levels of glycolysis metabolites, along with elevated levels of acetyl-CoA and carnitine in skeletal muscle of GAA-KO mice (Figure 1, Table S2) [17]. Nevertheless, Sato et al. (2016) reported an increase in many of the same metabolites in LOPD iPSC-derived cardiomyocytes and a possible disruption in β-oxidation based on a decrease in carnitine and oxidative stress affecting mitochondrial functionality (Figure 1, Table S2) [23]. Regarding ATP levels, Sato et al. (2016) found an increase, while studies like those by Meena et al. (2020) and Lim et al. (2015b) reported a reduction (Table S2) [17,18,23]. It is worth mentioning that this decrease in ATP production has been attributed to mitochondrial dysfunction rather than glucose depletion [18]. Based on this evidence, it is difficult to determine if nutrient deprivation occurs in PD and if that is the cause of the AMPK and mTORC1 signaling disruption.

Concerning cytoplasmic glycogen metabolism, paradoxically, an increase in the levels of proteins involved in its synthesis, like those of glycogen synthase and glycogenin, has been found in PD cells [25]. As for glycogen degradation, decreased glycogen phosphorylase activity has been documented (Figure 1) [25]. These alterations were recently confirmed by Canibano-Fraile et al. (2023) in murine models and human tissues related to glycogen phosohorylase and other proteins in such metabolism, like branching enzymes, UDP-glucose pyrophosphorylase and glucose transporter GLUT4 [26]. Through the network construction, AMPK was found to mediate the expression and phosphorylation of PGC-1α (peroxisome proliferator-activated receptor-gamma coactivator) [27], a molecular intermediate that has not been studied in PD. PGC-1α has been associated with glucose 6-phosphate accumulation (G6P). G6P acts as a glycogen synthase allosteric activator and inhibits genes that control glycogenolysis (e.g., glycogen phosphorylase and its activating kinase) [27]. This description agrees with evidence from skeletal and cardiac muscle of GAA-KO mice, in which the activity of glycogen phosphorylase is reduced, while G6P levels and glycogen synthase activity are elevated [15,25,28]. However, in the case of glycogen synthase, the authors explained such an alteration according to changes in the enzyme degradation rate rather than its expression [25].

Disruptions in glycogen metabolism may have consequences that remain unknown in PD. For example, a decrease in phosphorylase activity might increase the induction of glycogen-specific autophagy, called glycophagy [29]. This process has been proposed in PD based on the increased Stbd1 levels (starch binding domain 1) found in GAA-KO mice, which is the protein believed to transport glycogen inside the lysosome [30,31]. Moreover, recently, Stbd1 has been proposed as a potential target for therapy based on evidence of reduction in lysosomal glycogen accumulation in STBD1/GAA double knockout mice [31,32].

Another probable consequence is the hypoglycosylation of LAMP1 and LAMP2 (lysosomal-associated membrane protein) found in IOPD iPSC-derived cardiomyocytes (Figure 1, Table S1) [22]. Some authors explain that this could be due to the requirement of UTP and G6P for glycogen synthesis. Since this pathway is thought to be disrupted, these reactants may be used disproportionately and limit reactions that also require these molecules, like glycosyltransferase reactions in the Golgi apparatus [22]. Finally, substrate reduction therapy has been proposed through inhibition of either glycogenin or glycogen synthase as an alternative therapeutic approach [8].

### 2.4. PGC-1α Connects mTORC1 with Mitochondrial Biosynthesis and Function

**Figure 3:**
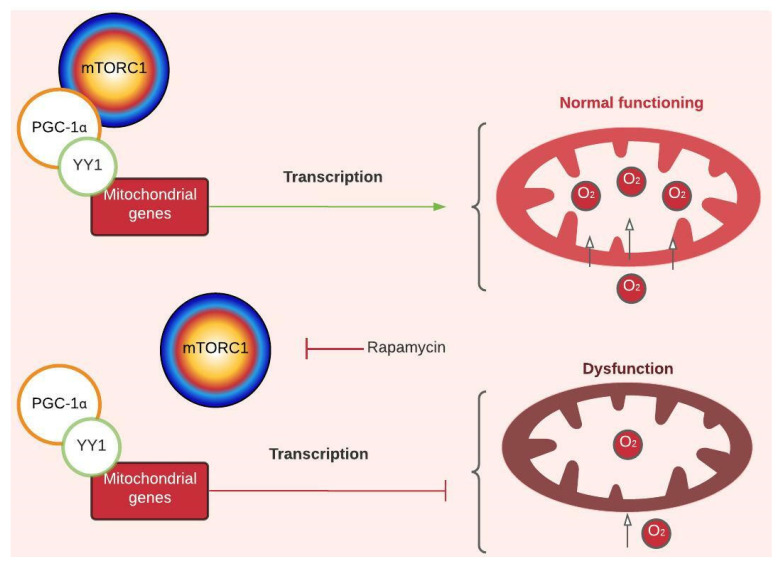
Regulatory mechanism of mTORC1-dependent mitochondrial gene transcription. Peroxisome proliferator-activated receptor-gamma coactivator (PGC 1-α). Transcription factor Yin Yang (YY1). The green arrow represents activation, and the red arrows represent inhibition. For more details, see Figure 1. Created with Lucidchart^®^ (https://www.lucidchart.com/pages/ accessed on 8 June 2023).

Mitochondrial dysfunction in PD is inferred from physiological and morphological changes, like lower oxygen intake and ATP production, which have been described in muscle tissue from GAA-KO mice; larger mitochondria with altered form; and the presence of inclusions observed in muscular tissue of PD patients [9,18,33]. Such dysfunction has been attributed to increased calcium levels inside the mitochondria associated with CACNB1 (voltage-dependent L-type calcium channel subunit beta-1) upregulation [19]. However, through network enrichment, two new nodes that link the expression of mitochondrial genes and their respiration capability with mTORC1 were found: PGC-1α and YY1 (transcription factor Yin Yang) (Figure 1). Cunningham et al. (2007) demonstrated that YY1 binds to promoters of mitochondrial genes, and PGC1-α acts as its transcriptional coactivator in an mTORC1-dependent manner (Figure 1 and Figure 3). Researchers have observed that treating skeletal muscle with rapamycin (mTORC1 inhibitor) not only downregulates mitochondrial genes but also decreases the amount of oxygen that enters the mitochondria and lactate production without affecting ATP levels [34]. PD evidence agrees with less oxygen intake and lactate production (Figure 1) [17,18], but Meena et al. (2020) also reported lower production of ATP in skeletal muscle of GAA-KO mice. This mechanism offers an alternative explanation for mitochondrial dysfunction in PD. In addition, YY1 has been implicated in activation of muscular stem cells (or muscle satellite cells (SCs)) due to its role as a positive upstream regulator of not only mitochondrial genes but also of glycolysis-associated genes through stabilization of HIFα [9]. This is interesting, considering that skeletal muscle of PD patients has shown a weak regenerative response related to an SC activation impairment, which might be reversible and even potentiated as a therapeutic target in PD [35,36,37,38]. Furthermore, there is evidence of *GAA* gene expression changes during early myogenesis [39]. All this information points out the importance of understanding the interplay of different cellular pathways not only in muscle fibers but also in other cell types involved in the preservation of muscle function and structural integrity.

### 2.5. MCOLN1, Calcineurin, and Keap1 Couple Oxidative Stress and Antioxidant Response with Autophagy

Lim et al. (2015b) mentioned that damaged mitochondria can become a source of reactive oxygen species (ROS). In this regard, evidence has shown elevated ROS levels in GAA-/- myotubes and muscle cell cultures of LOPD patients, in addition to an increase in oxidative stress markers like GSSG and total glutathione [23].

**Figure 4:**
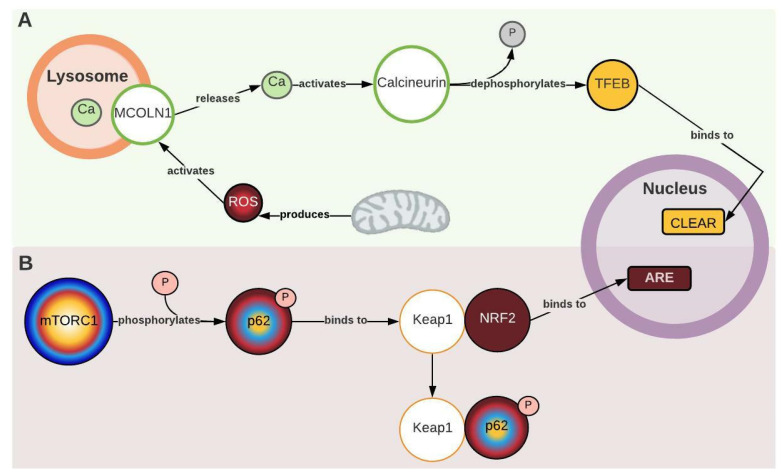
Mechanisms that couple oxidative stress and antioxidant response with autophagy. (**A**) Mucolipin-1 (MCOLN 1). Transcription factor EB (TFEB). CLEAR (coordinated lysosomal expression and regulation). (**B**) Kelch-like ECH-associated protein 1 (Keap1). Nuclear factor erythroid 2-related factor 2 (NRF2). For more details, see Figure 1. Created with Lucidchart^®^ (https://www.lucidchart.com/pages/ accessed on 8 June 2023).

During network construction, two new nodes that act as ROS sensors and trigger an autophagic response were identified: MCOLN1 (mucolipin-1) and calcineurin. According to Zhang et al. (2016), MCOLN1 releases calcium from the lysosome to the cytoplasm when it detects ROS produced by the mitochondria. Then, the released calcium activates calcineurin, which dephosphorylates TEFB (transcription factor EB). Dephosphorylated TFEB translocates to the nucleus and promotes the transcription of autophagic genes to break down the damaged mitochondria (Figure 1 and Figure 4A) [40]. This description coincides with what has been evidenced in PD. Lim et al. (2015) found an excess of cytoplasmic calcium, along with a significant increase in PINK1 and PARK2 levels (proteins involved in the signaling of damaged mitochondria) and accumulation of ubiquitinated mitochondria (Figure 1). All this evidence suggests increased mitochondria-specific autophagy, known as mitophagy [18]. Therefore, this mechanism could explain how oxidative stress may be part of the autophagic response and the increase in intracellular calcium in PD. It is worth noting that despite its involvement in autophagy, TFEB overexpression does not necessarily exacerbate AuP-BU. Both TFEB and TFE3 (transcription factor E3) have been contemplated as therapeutic targets for PD because they promote lysosomal exocytosis and decrease glycogen accumulation [8,41,42].

In summary, with the information gathered in the network, it seems, as mentioned in the previous sections, that mTORC1 disruption alters mitochondrial function, triggering ROS production, which, in turn, exacerbates autophagy, leading to a deleterious cycle in PD. In fact, recent reports showed that coupling ERT with antioxidant therapy improves recombinant enzyme activity and Aup-BU [43,44], supporting our findings and pointing out that mitochondrial function and oxidative stress might be aspects that deserve further research. 

Another molecular element identified through the network that couples antioxidant response to autophagy is Keap1 (Kelch-like ECH-associated protein 1). Katsuragi et al. (2016) described two pathways by which Keap1 mediates an antioxidant response: the canonical pathway, which is activated by the presence of ROS, and the non-canonical pathway, which involves p62. In the non-canonical pathway, p62 is phosphorylated by mTORC1 and competitively binds to Keap1, which allows for the translocation of NRF2 to the nucleus, where it triggers an antioxidant response (Figure 1 and Figure 4B). This pathway is hyperactivated in autophagy-deficient murine liver cells and hepatocellular carcinoma tissues, partly due to the accumulation of p62 phosphorylated by mTORC1 [45]. In PD, Raben et al. (2008) described an accumulation of non-phosphorylated p62 in GAA-KO mice [46], and Sato et al. (2016) demonstrated a decrease in the antioxidant response (ARE) mediated by NRF2 (Figure 1, Table S1) [23]. This seems to be contradictory to the “non-canonical” pathway description, but considering that the basal activity of mTORC1 is decreased in PD, this would cause an inhibition of the mechanism due to the lack of p-p62, which may exacerbate oxidative stress effects in the pathology.

### 2.6. PERK1 Couple ER Stress with Protein Synthesis Inhibition

ER stress occurs when disruptions or modifications in protein folding occur. This can be caused by mutations, inhibition of protein glycosylation, and alterations in intracellular calcium stores [47]. ER stress mediates apoptosis in many lysosomal storage diseases, such as neuronal ceroid lipofuscinosis, Gaucher disease, and Niemann–Pick type C [47]. Shimada et al. (2011) observed all three responses triggered by ER stress in fibroblasts from PD patients (carrying the c.546G > T mutation in the *GAA* gene): unfolded protein response (UPR), as evidenced by increased activity of stress-sensing kinases IRE1α and PERK, as well as by the levels of the BiP/Grp78 chaperone; degradation of proteins associated with the endoplasmic reticulum (ERAD), as indicated by decreased levels of mature GAA due to its degradation by the proteasome; and, finally, apoptosis mediated by p38, as demonstrated by increased levels of p-p38 (Figure 1 and Figure 2C) [47].

**Figure 5:**
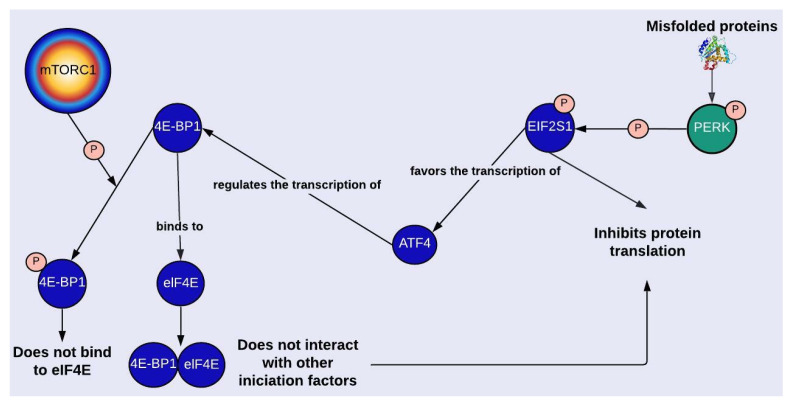
mTORC1-dependent and independent regulation of protein synthesis. Protein kinase RNA-like endoplasmic reticulum kinase (PERK). Eukaryotic translation initiation factor 2 subunit 1 (EIF2S1 o eIF2α). Activating transcription factor 4 (ATF4). Eukaryotic translation initiation factor 4E-binding protein 1 (4E-BP1). Eukaryotic translation initiation factor 4E (eIF4E). For more details, see Figure 1. Created with Lucidchart^®^ (https://www.lucidchart.com/pages/ accessed on 8 June 2023).

S6K (ribosomal S6 kinase) and 4E-BP1 (eukaryotic translation initiation factor 4E-binding protein 1) are involved in the regulation of protein synthesis; 4E-BP1 interacts with eIF4E (eukaryotic translation initiation factor 4E), and S6K phosphorylates S6 (ribosomal protein S6) [48]. The activation of both proteins depends on mTORC1-mediated phosphorylation, which could explain decreased p-4E-BP1 and p-S6K levels in PD (Figure 1 and Table S1) [15,17]. In general, these proteins are evaluated in PD investigations as markers of mTORC1 activity, but there is another mechanism related to 4E-BP1 and independent of mTORC1 by which protein synthesis is regulated. One of the transcription factors that regulates the expression of 4E-BP1 is ATF4 (activating transcription factor 4), whose transcription is favored when EIF2S1 (eukaryotic translation initiation factor 2 subunit 1) is phosphorylated. Through the enrichment of the network, it was possible to identify PERK1 as the enzyme responsible for phosphorylating EIF2S1 (Figure 1 and Figure 5). Thus, endoplasmic reticulum stress can repress protein synthesis through UPR by activating EIF2S1, which is known for inhibiting general protein translation, and through the transcription of 4-EBP1.

Nevertheless, the evidence in PD regarding EIF2S1 phosphorylation is contradictory. According to Lim et al. (2017), there is an increase of p-EIF2S1 (p-eIF2α), which could be explained by the increment in active PERK1 [15]. But Lim et al. (2018) reported that p-EIF2S1 levels are decreased in GAA-KO mice, which agrees with the hypothesis of protein synthesis increase as a mechanism to compensate for the increment in protein degradation through autophagy and the proteasomal system caused by mTORC1 inhibition [13,28,49]. In line with this, Lim et al. (2018) found higher activity levels of the proteolytic enzyme CAPN2 and of proteasome subunits (Figure 1) [13]. Overall, what seems to be clear is that an imbalance between protein synthesis and degradation causes net protein loss and a reduction in muscle function, which may contribute to the deterioration of skeletal muscle in PD [13].

### 2.7. Mtorc1 and Autophagic Lysosome Reformation (ALR)

**Figure 6:**
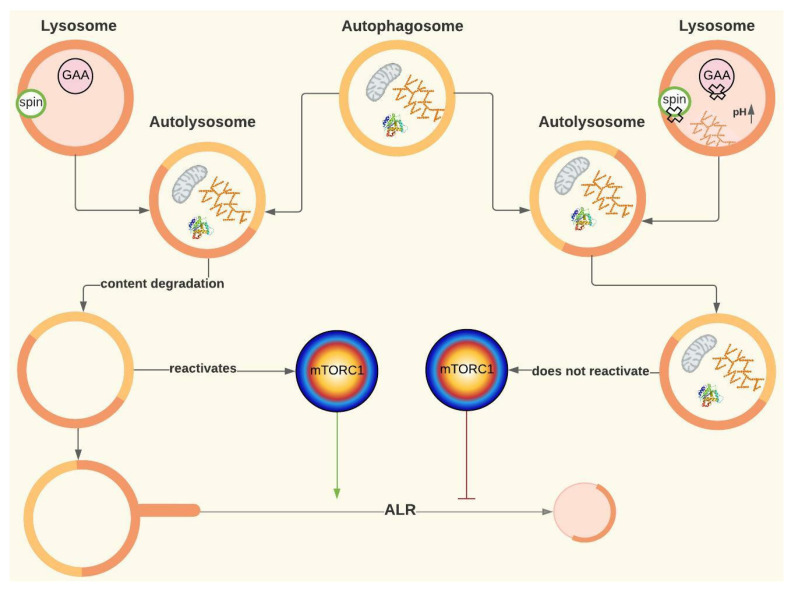
ALR and its possible role in Pompe disease. Spinster (spin). The green arrow represents activation, and the red arrow represents inhibition. For more details, see Figure 1. Created with Lucidchart^®^ (https://www.lucidchart.com/pages/ accessed on 8 June 2023).

It is known that to initiate autophagy, mTORC1 must be inhibited. Yu et al. (2010) found that in rat kidney cells, mTORC1 was reactivated after a 12 h nutrient deprivation period (Figure 1 and Figure 6). mTORC1 reactivation triggers the initiation of ALR, a mechanism by which cells recover the lysosomes that were “consumed” during autophagy. ALR is an evolutionarily conserved process that governs nutrient sensing and lysosome regeneration following starvation-induced autophagy, thus maintaining lysosomal homeostasis. This process is mediated by proteins such as mTORC1 and Spinter (spin), as well as by the dissociation of GTPase Rap7 and the subsequent overexpression of Rab7, among other proteins. Once the autolysosomes have degraded all their content, tubules arise from them, forming vesicles that later mature into lysosomes (Figure 1 and Figure 6) [50]. This pathway has been evidenced in many cell lines, although the activation kinetics vary between organisms.

Since reactivation of mTORC1 depends on autolysosomal content degradation, ALR might be disrupted in PD [51]. Although a disruption in lysosomal regeneration has not been described in this disease, it has been evidenced in cells of patients with other lysosomal storage diseases, such as Fabry disease, Scheie syndrome and aspartylglucosaminuria [50]. In fact, Nanayakkara et al. (2022) have demonstrated that defects in lysosomal degradation capacity occurring in other lysosomal storage disorders lead to a defective ARL process, which would contribute to the pathological cascade in these diseases as the origin of the Aup-BU [52].

Yu et al. (2010) treated rat kidney cells with rapamycin and discovered that not only did the lysosomes not regenerate, but regions with enlarged autolysosomes, which persist for a long time, were also formed, similar to AuP-BU. These authors also inhibited the proteolytic capacity of the lysosome, obtaining a similar result to rapamycin treatment [50]. Rong et al. (2011) observed the same result by inhibiting spin (Spinster), which is thought to be a H +/carbohydrate symporter. The hypothesis is that spin inhibition blocks the flow of H+ from the lysosome, leading to changes in the optimal pH at which lysosomal enzymes work [51]. In fact, authors like Lim et al. (2015a) suggest that ALR disruption could be involved in PD based on the notorious similarities, at least in terms of AuP-BU, between spin inhibition and GAA deficiency [19,51]. Thus, for PD, the hypothesis is that if glucose is also transported by spin, the lack of glucose inside the lysosome caused by GAA deficiency may affect H+ flow and have a similar effect to that of spin inhibition (Figure 2D and Figure 6) [19].

### 2.8. Perspectives and Limitations

It is worth noting that due to the theoretical nature of the network, it is important to address the proposed mechanisms and molecular elements from an experimental perspective that confirms their participation in PD.

The incorporation of evidence outside the context of the disease may enrich future research in PD, since it considers different approaches to those traditionally studied. In addition, the network’s design facilitates the interpretation of the information collected and helps to visualize knowledge gaps that contribute to the proposal of new questions, such as:

- What are the activation kinetics of mTORC1 in PD cells? Since AKT has been shown to inhibit mTORC1 in fibroblasts from PD patients and insulin regulates its pathway [53] (Figure 1 and Figure 3 and Table S1), what is the role of hormones in PD?
- Does hypoglycosylation due to alteration in glycogen metabolism contribute to endoplasmic reticulum stress and AuP-BU?
- Why is autophagy central to skeletal muscle and not cardiac pathophysiology?
- All this adds to the role of some systemic events that were not considered in this work, like the contribution of inflammatory processes and immune response suggested by proteomic profiles (Tables S1 and S2) [54].

## 3. Materials and Methods

### 3.1. Search of Experimental Evidence in PD

In order to identify the main molecular elements and cellular processes of potential relevance to understand the relationship between GAA deficiency and the pathologic cascade, a review of the experimental evidence on the subject was carried out.

**Figure 7:**
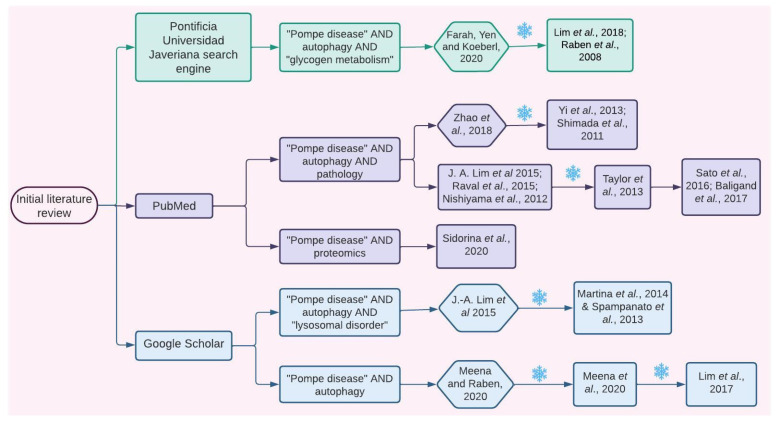
Information search. Explicative flow chart showing how the articles from which the information for the initial network design was extracted were selected. Hexagons represent review articles, and squares represent original articles. Each number corresponds to an article in the reference section. Green color represents the search performed using the Universidad Javeriana search engine, purple color represents the PubMed search, and the blue color represents the Google Scholar search. The snowflake symbol specifies times when the “snowball” search strategy was used [1,7,13,15,17,18,19,22,23,25,28,29,30,41,42,46,47,53,54].

For this purpose, the Pontificia Universidad Javeriana institutional search engine, Google Scholar, and PubMed were used. The search keywords are specified in Figure 7, and only articles published from 2006 to 2020 were considered. Since literature about Pompe disease is broad and in order to include the most recent evidence, initially, a set of the four most recent reviews and five primary research articles was selected. This selection was made taking into account articles that focused on general or specific mechanisms associated with PD pathophysiology and that provided details about molecular intermediaries. Articles were included only if they detailed evidence related to proteins; metabolites; or other kind of molecules whose expression, quantity, and/or activity levels were altered in samples from PD patients and/or PD models such as GAA-KO mice or patient-derived induced pluripotent stem cells (iPSC). Information regarding molecular interactions was confirmed and further characterized by performing an in-depth search focused on finding original articles using the “snowball” method (Figure 7).

**Figure 8:**
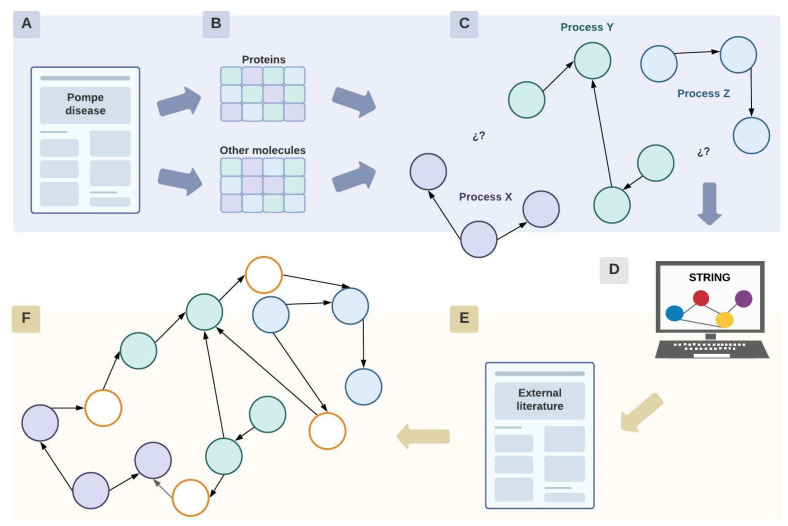
Network construction. Diagram representing the process of construction of the network, where circles represent proteins or other molecular elements. Briefly, initially information search was performed in PD literature (**A**), proteins and other moleculaes involved were included in a database (**B**) to further represent the relationship described in an initial network (**C**) whose gaps were filled with information outside PD context (**D**–**F**). Each color implies a different cellular process or pathway. White nodes represent proteins or molecular elements that were found during the enrichment process of the network with STRING and literature external to the context of the pathology. Created with Lucidchart^®^ (https://www.lucidchart.com/pages/ accessed on 8 June 2023).

The obtained information was organized in two matrices (Figure 8A,B): one for proteins only (Table S1) and one for other kinds of molecules (Table S2).

### 3.2. Network Construction

#### 3.2.1. Initial Network Design

The initial network design was diagrammed with Lucidchart^®^ based on these two matrices, specifically considering the data related to the function and experimental evidence of each molecular element in PD (Tables S1 and S2).

The network consists of nodes representing molecular elements connected by edges that represent functional interactions. It was constructed manually in an unweighted manner, which means that the edges do not have a value assigned to them. Each node has a symbol that specifies how that particular element is altered in PD, along with a color code that organizes the nodes in specific cellular processes (Figure 8C). Physical interactions and organelles are represented, but only when the literature mentioned their relevance for the comprehension of the pathology. These interactions between proteins or with certain organelles are illustrated by the union of continuous nodes or with the corresponding organelle.

Apart from identifying the interactions between molecular elements within the context of the disease, this network was crucial to acknowledging the cellular processes with few or without molecular intermediates that establish a link between them (Figure 8C).

#### 3.2.2. Network Enrichment

Once the initial network was finished, it was enriched using information from the STRING database for protein interactions to fill the gaps (Figure 8D). For these, proteins considered as “origin nodes” within the network were used as inputs in STRING. Here, “origin nodes” refer to proteins whose signaling pathways are the starting point of signaling pathways or cellular processes. This search aimed to increase the connectivity of the initial network and to investigate in-depth interactions that may be relevant for the comprehension of the pathologic cascade.

A STRING search was conducted exclusively for human proteins considering evidence related to the literature, coexpression, and experimental data. Protein selection was based on the interaction score, which represents the confidence of the interaction. Therefore, this score was established according to the STRING parameters: between 0.4 (intermediate) and 0.9 (very high) (Figure 8D).

STRING displays a network centered around the proteins used as an input in the search engine. The number of maximum interactions was settled within a range of 5–20 interactions. The aim was to find proteins whose interactions were close to the input proteins in order to identify direct interactions.

To understand how the proteins obtained with STRING might be involved in the pathologic cascade, a review of their function was carried out by consulting literature external to PD (Figure 8E). Proteins were included in the network if they complemented the information reported in PD (Figure 8F).

## 4. Conclusions

The network not only allows for an overview of the current knowledge of the molecular bases of PD but also complements it with sources external to the context of the pathology. Through its analysis, it was possible to identify the central role of AMPK and mTORC1 in the cascade of pathophysiological events. Moreover, the network provides several intermediaries that could be the object of future experimental studies, such as PGC-1α and spin, among others. On the other hand, the interplay between cytosolic and lysosomal glycogen metabolism in PD was identified as a knowledge gap that has been poorly addressed. In addition, during the construction of the network, ALR was identified as a process that could explain how GAA deficiency generates an autophagic alteration, although it is unclear whether this alteration responds to mechanisms associated with global lysosomal dysfunction or specific aspects of the mutated enzyme. Finally, this theoretical approach provided a clear view of current knowledge gaps, as well as a perspective that allowed for the identification of novel potential intermediates and mechanisms. All these open the possibility of generating new hypotheses and questions that can be addressed in future experimental studies in order to improve our understanding of Pompe disease, which is essential for developing better therapeutic strategies.

## Data Availability

The data presented in this study are available in the Supplementary Materials.

## Supplementary Materials

The supporting information can be downloaded at: https://www.mdpi.com/article/10.3390/ijms241512481/s1.
